# Rubber and plantain intercropping: Effects of different planting densities on soil characteristics

**DOI:** 10.1371/journal.pone.0209260

**Published:** 2019-01-09

**Authors:** Erasmus Narteh Tetteh, Akwasi Adutwum Abunyewa, Henry Oppong Tuffour, Joseph Nketiah Berchie, Patricia Pinamang Acheampong, Kwame Twum-Ampofo, Evans Dawoe, Vincent Logah, Olivia Agbenyega, Stella Ama Ennin, Isaac Nunoo, Caleb Melenya, Eric Owusu Danquah, Victor Rex Barnes, Samuel Tetteh Partey

**Affiliations:** 1 Crops Research Institute, Council for Scientific and Industrial Research, Kumasi, Ghana; 2 Department of Agroforestry, Kwame Nkrumah University of Science and Technology, Kumasi, Ghana; 3 Department of Crop and Soil Sciences, Kwame Nkrumah University of Science and Technology, Kumasi, Ghana; 4 CGIAR Research Program on Climate Change, Agriculture and Food Security, International Crops Research Institute for the Semi-Arid Tropics, Bamako, Mali; RMIT University, AUSTRALIA

## Abstract

Two field experiments were conducted at Ellembelle and Jomoro districts in the Western region of Ghana where rubber cultivation is a predominant farming activity. The objective of the study was to assess the effect of rubber and plantain intercropping systems on selected soil properties. The experiment was arranged in a randomized complete block design (RCBD) with 3 replications. The treatments were the sole crop rubber (R), sole crop plantain (P) and three intercrop systems comprising an additive series of plantain: one row of plantain to one row of rubber (PR), two rows of plantain to one row of rubber (PPR) and three rows of plantain to one row of rubber (PPPR). Generally, agroforestry systems improved the soil hydraulic properties considerably, with the highest cumulative infiltration rates of 5.16 and 8.68 cm/min observed under the PPPR systems at the Ellembelle and Jomoro sites, respectively. Microbial biomass C (C_mic_), N (N_mic_) and P (P_mic_) was significantly improved (P < 0.05) under the agroforestry than the monocrop systems. The C_mic_, N_mic_ and P_mic_ values were highest under the PPPR system at both Ellembelle (C_mic_, = 139.9 mg/kg; N_mic_ = 36.26 mg/kg and P_mic_ = 87.6 mg/kg) and Jomoro (C_mic_ = 78.7 mg/kg; N_mic_ = 80.3 mg/kg and P_mic_ = 3.45 mg/kg) sites.

## Introduction

Natural rubber production in Africa is about 5% of the world’s production, with Cote d’lvoire being the lead producer (300,000 tonnes per annum), followed by Liberia. The current production capacity of Ghana, which is 15,000 tonnes per year is anticipated to reach 50,000 tonnes by the year 2020 [1]. With the vision to spearhead economic empowerment through rubber cultivation for sustainable rural community development to alleviate poverty, the introduction of the Rubber tree out-grower Scheme was introduced in 1995. As a result, rubber tree cultivation has become a fast growing enterprise in Ghana, and most farmers, especially in the high rainforest zone (i.e., in the Western region) of the country have shifted to rubber cultivation as a sustainable source of income due to the ready market after latex production begins.

The major challenge in the production of rubber worldwide, is the long gestation period (six years), which deprives the farmers of sustainable income during the immature phase, as well as, uneconomic land size (small-holder farm sizes) available for plantation establishment [2]. In consequence, farmers resort to intercropping rubber with short duration annual and perennial crops to offset the six years of income gap between the time of establishment and latex production. This practice is an evidence of studies which have reported that rubber agroforestry reduces the cost of the management of the plantation by ensuring early generation of revenue to the farmer in the immaturity period of the rubber [3–5]. However, this practice, had been perceived in the negative sense in respect of competition for both below and above ground resources [6, 7] such as solar radiation, water, nutrients etc. for growth and development. Few studies have suggested that rubber agroforestry systems improve soil properties [8], and rate of growth of rubber [9].

Despite the several socio-economic benefits of rubber, such as diversification of agricultural products, increase in income levels for farmers, employment opportunities in the farming communities, foreign exchange earnings, and enhancement of women economic emancipation [1], there are fears that increased production will pose a great threat to food security in the rubber growing areas on the globe. Hence, more attention is needed to complement the nexus between rubber development and food security. The current study was, therefore conducted to evaluate the effect of plantain density in rubber and plantain intercropping systems on soil characteristics.

## Materials and methods

### Description of study areas

**Fig 1:**
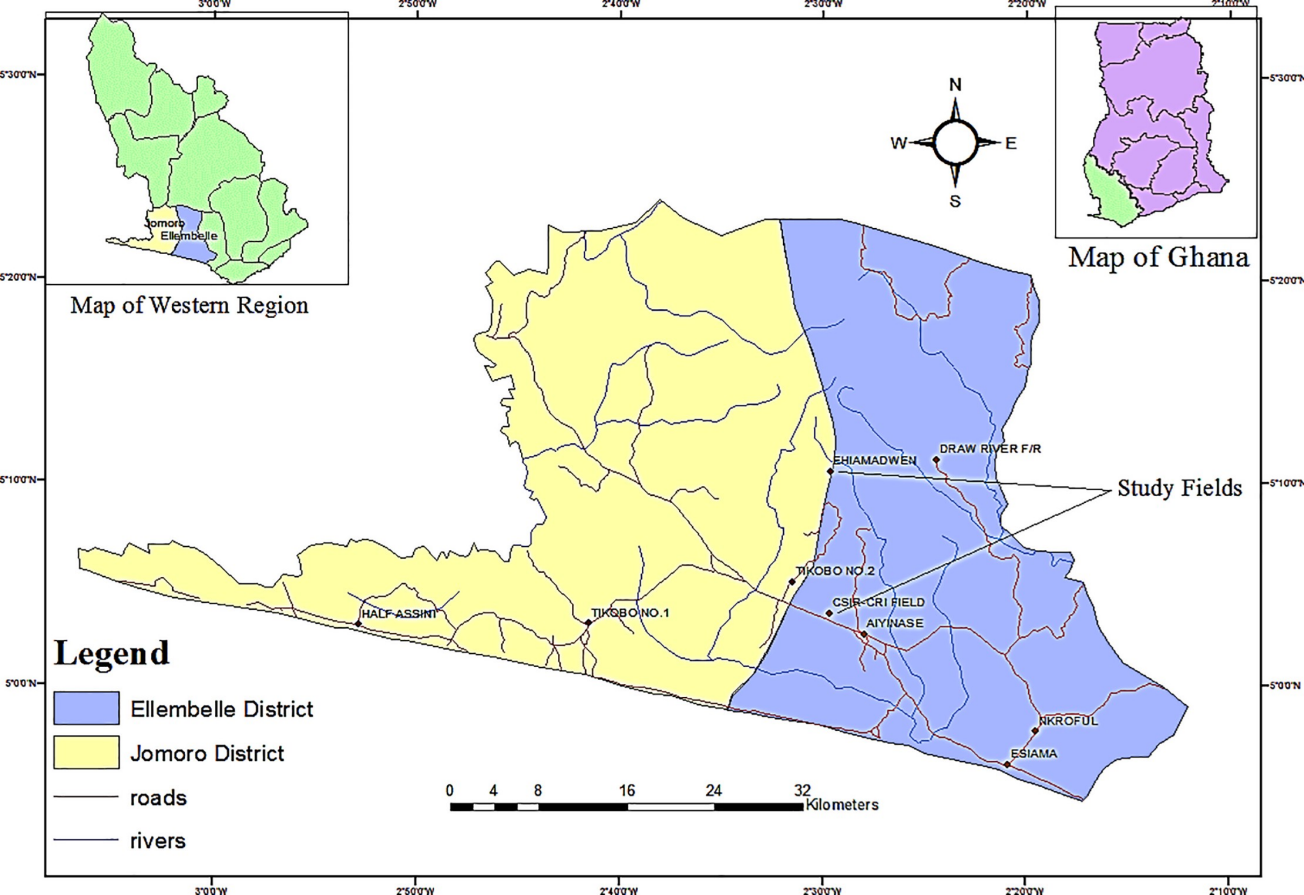
Map of Ellembelle and Jomoro districts showing the study fields.

Field trials were conducted at two different locations in the Western region of Ghana where rubber production is prevalent. The locations were Crops Research Institute of the Council for Scientific and Industrial Research (CSIR-CRI) stations at Aiyinasi in the Ellembelle district (between longitudes 2° 05’ W and 2° 35’ W and latitude 4° 40’ N and 5° 20’ N) and Tikobo No. 2—Ehiamadwen in the Jomoro district (between latitudes 4° 80’ N and 5° 21’ N and longitudes 2° 35’ W and 3° 07’ W) (Fig 1). The Ellembelle district falls within the wet semi-equatorial climatic zone of the West African Sub-region, and Axim belt, with a semi-deciduous rainforest vegetation where there is rainfall almost throughout the year. The maximum mean monthly rainfall ranges between 26.8 mm to 46.6 mm; mean temperature is about 29.40°C with the mean monthly temperature variation between 4–5°C. The relative humidity is about 90% during the night and about 75% during the afternoon, especially in June and July [10]. The predominant soil types are Ferric Acrisols and Dystric Fluvisols [11]. The Jomoro district, which has a large traditional agrarian sector (nearly 60% of total labour force) is located in the South western part of the Western Region of Ghana [10]. The District is reportedly the wettest part of Ghana [12], with a monthly mean temperature of 26°C, relative humidity of about 90% at night and 75% in the afternoon [10].

### Field layout and design

**Fig 2:**
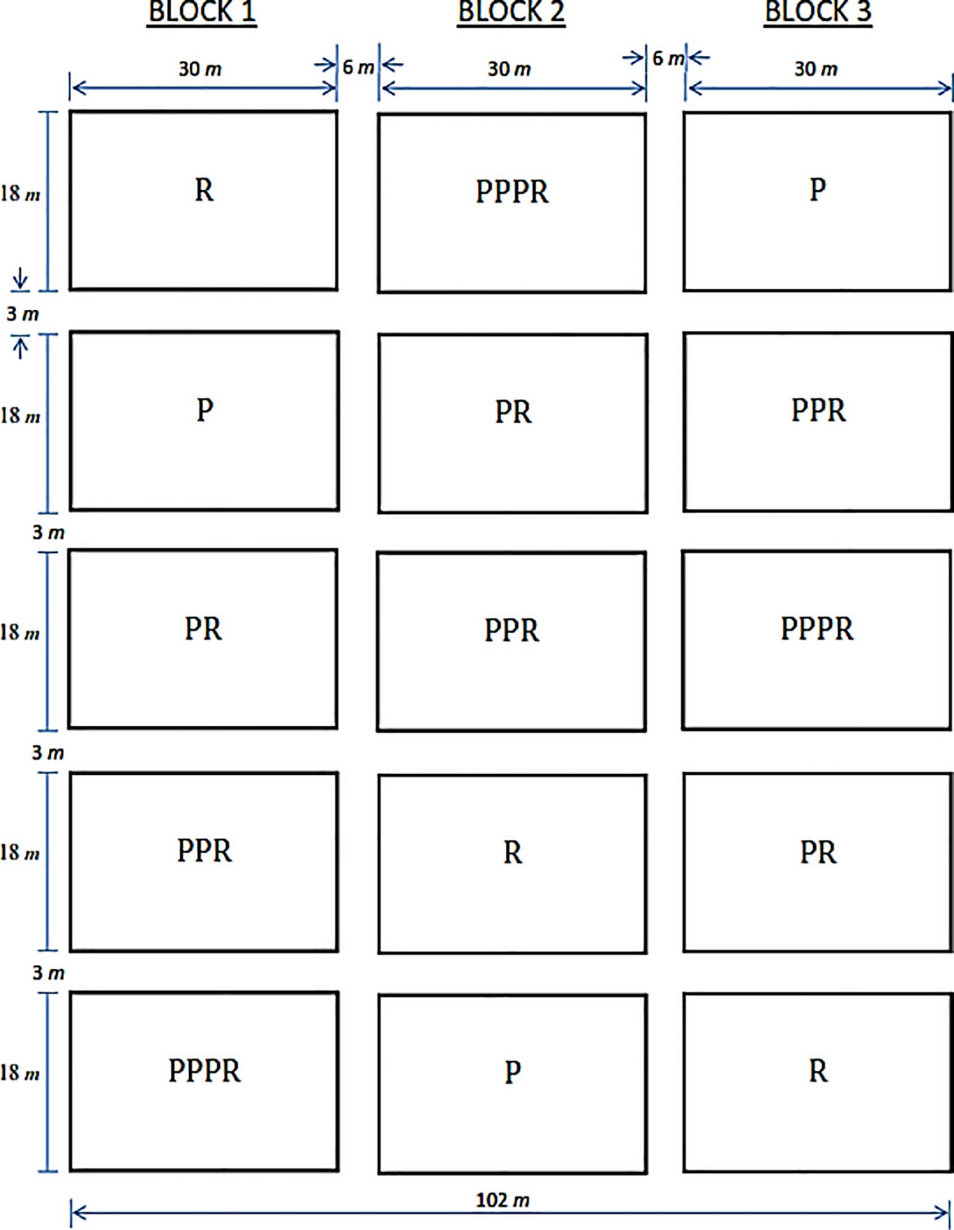
Field layout showing the randomization of the treatments.

The study field at Aiyinasi (05° 03.517’ N and 002° 29.782’ W) in the Ellembelle District was a cleared old and abandoned rubber plantation with regenerated tree species, whereas the trial at Tikobo No. 2 –Ehiamadwen 05° 10.234’ N and 002° 28.768^’^ W in the Jomoro district was conducted on an abandoned regenerated oil-palm plantation. The field size was 102 m x 102 m for each experimental set-up. The experiment comprised five treatments: sole crop rubber (R), sole crop plantain (P) and three intercrops consisting of an additive series of plantain, one row of plantain (PR), two rows of plantain (PPR) and three rows of plantain (PPPR) between two rows of rubber arranged in a Randomized Complete Block Design with three replications (Fig 2).

**Fig 3:**
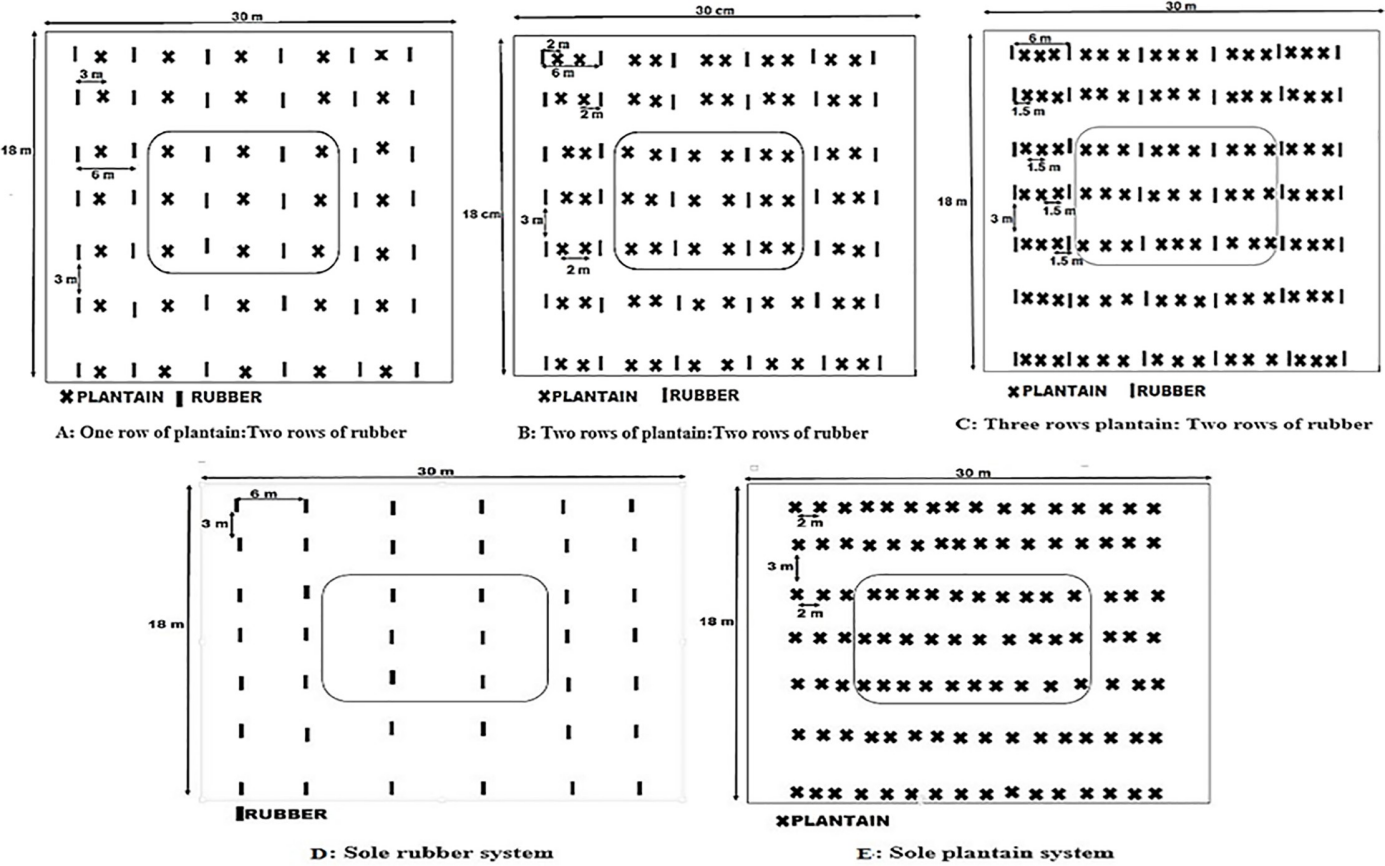
Plant stands of the rubber, plantain and the intercropping systems. The marked plants were the selected plants for the trial.

The rubber clone GT1 and the false horn plantain variety (locally referred to as “Apantu pa”) were used for the study. Planting density of rubber was 555 plants/ha in both sole and intercrops, 555 plants/ha for PR, 1111 plants/ha for PPR, 1666 plants/ha for PPPR, and 1666 plants/ha for P. In all intercrop treatments, rubber was planted at a spacing of 3 m within, and 6 m between rows, whereas 3 x 2 m spacing was used for the planting of sole plantain crops. Intra-row spacing for both rubber and plantain was kept constant at 3 m, whilst the inter-row spacing was varied in keeping with the number of plantain rows as 3 m, 2 m and 1.5 m in PR, PPR and PPPR, respectively (Fig 3). Rubber was planted simultaneously with the plantain after lining and pegging of the respective treatment plots. Fertilizer (NPK 15:15:15) was applied at a rate of 100 g/plant for plantain in a ring of 50 cm radius around each plant at 30 days after planting, and 200 g/plant for rubber at 180 days after planting by spreading/broadcasting in furrows within the range of the spread of the canopy and covered immediately.

### Soil and plant tissue sampling and analyses

Soil samples were collected at random at 0–15 cm, 15–30 cm and 30–60 cm depths at 10 cores per depth (30 cores per plot) from the plots in each block and were bulked as three composite samples representative of the block for initial characterization before the treatments were imposed. The final soil samples were also taken from each plot at 10 cores per depth (30 cores per plot). These samples were analyzed after they were air–dried, crushed and sieved through a 2 mm sieve for the determination of particle size distribution [13], bulk density, moisture content, total nitrogen [14], available phosphorus [15] and exchangeable potassium. Soil sampling for bulk density and moisture content were measured at 48 h after rainfall, when the soil was assumed to be at field capacity. Microbial biomass [16] was determined in samples from the 0–15 cm using the field moist or fresh soil. Field infiltration studies were conducted using the single ring infiltrometer. Data was also taken on infiltration amount, infiltration rate and sorptivity. Plant tissue analyses of the rubber and plantain was done for OC, N, P and K at the Jomoro study site.

### Statistical analysis

The data obtained from the two field trials were subjected to analysis of variance (ANOVA) using GenStat (12^th^ edition). Mean separation was done using the standard error of difference (SED) at a significance level of 0.05. The Duncan’s Multiple Range Test was used to identify pairs of means that differed significantly.

## Results

As expected, bulk density at both Ellembelle and Jomoro sites increased with soil depth. Soil texture at the Ellembelle site was found to be sandy loam (SL) in the 0–15 and 15–30 cm depths, whereas, for the Jomoro site, textures ranged from sandy loam (0–15 cm), sandy clay loam (15–30 cm) to clay loam (30–60 cm). Furthermore, hard pan was encountered along the soil profile, especially within the 30–60 cm depth in both sites. This is evidenced by the high clay contents down the profile, especially, at the Jomoro site (Table 1).

**Table 1:** Initial soil physical properties at the Ellembelle and Jomoro sites before treatment applications.

*Soil property	Ellembelle site			Jomoro site		
	0–15 cm	15–30 cm	30–60 cm	0–15 cm	15–30 cm	30–60 cm
Bulk density (Mg m^-3^)	1.35	1.58	HP	1.24	2.39	HP
Moisture content (%)	9.08	7.53	HP	1.65	12.17	HP
Sand (%)	75.04	73.89	76.29	56.00	48.00	45.00
Silt (%)	14.09	16.96	14.01	25.12	21.12	17.08
Clay (%)	10.87	9.15	9.70	18.88	30.88	37.92
Texture	SL	SL	HP	SL	SCL	CL
Available P (mg/kg)	3.80	4.01	4.78	-	-	-
Total N (%)	0.30	0.22	0.11	0.15	0.08	0.07
SOC (%)	2.70	0.96	0.70	1.72	0.89	0.74
K (cmol_c_/kg)	0.22	0.15	0.09	0.18	0.15	0.15
pH (1:2.5)	4.21	4.50	4.52	5.02	4.86	4.59
C_mic_ (mg/kg)	13.03	-	-	69.10	-	-
N_mic_ (mg/kg)	5.17	-	-	29.22	-	-
P_mic_ (mg/kg)	16.79			3.74	-	-

SL = Sandy loam; SCL = Sandy clay loam; CL = Clay loam; HP = Hard pan; ECEC = Effective Cation exchange capacity; TEB = Total exchangeable bases; C_mic_ = microbial biomass carbon; N_mic_ = Microbial biomass nitrogen; P_mic_ = Microbial biomass phosphorus

*Samples were bulked together and subsampled for the analyses

### Soil hydro-physical properties

Summary of the results on soil hydro-physical properties are presented in Table 2. There were no significant differences (P > 0.05) in bulk density across all treatments and depths (Table 2). Due to the presence of hard pan and high clay content (Table 1), bulk density and moisture content analyses were not measured at 30–60 cm depth at the Jomoro experimental site. Higher bulk density values were observed 15–30 cm depth than the 0–15 cm depth. The cropping systems with plantain, either as sole crop or inter crop had lower bulk density values in the 0–15 cm depth. On the contrary, a reverse pattern was observed in the 15–30 cm depth, high bulk density values were recorded in systems with plantain as sole or inter crops.

**Table 2:** Soil hydro-physical properties under rubber and plantain cropping systems at the Ellembelle and Jomoro sites.

Treatments	Depth (cm)	MC (%)	BD (kg/M^3^)	I (cm)	i (cm/min)	S (cm/min^1/2^)	K_o_ (cm/min)
**Ellembelle site**							
**P**	0–15	8.02^a^	1.40^a^	101.10^b^	3.37^b^	9.38^b^	20.00^b^
**R**	0–15	5.59^a^	1.72^a^	2.50^e^	0.08^e^	1.10^d^	0.50^d^
**PR**	0–15	5.59^a^	1.47^a^	40.70^d^	1.36^d^	4.14^c^	7.70^c^
**PPR**	0–15	7.64^a^	1.38^a^	85.70^c^	2.89^c^	8.76^b^	16.90^b^
**PPPR**	0–15	7.87^a^	1.49^a^	154.80^a^	5.16^a^	19.03^a^	38.00^a^
**SED (5%)**		NS	NS	15.36	0.35	1.69	4.42
**CV (%)**		28.50	8.90	24.40	16.90	24.40	32.60
**P**	15–30	6.00^c^	1.59^a^	-	-	-	-
**R**	15–30	5.72^c^	1.45^a^	-	-	-	-
**PR**	15–30	6.21^c^	1.57^a^	-	-	-	-
**PPR**	15–30	7.39^b^	1.46^a^	-	-	-	-
**PPPR**	15–30	10.94^a^	1.72^a^	-	-	-	-
**SED (5%)**		0.95	NS	-	-	-	-
**CV (%)**		16.00	6.50	-	-	-	-
**Jomoro site**							
**P**	0–15	19.60^a^	1.12^a^	98.00^b^	6.21^b^	1.64^b^	0.86^b^
**R**	0–15	20.30^a^	1.17^a^	53.00^c^	3.90^c^	0.88^c^	0.39^b^
**PR**	0–15	18.20^a^	1.15^a^	57.00^c^	3.57^c^	0.95^c^	0.51^b^
**PPR**	0–15	19.00^a^	1.26^a^	182.00^a^	8.23^a^	3.03^a^	2.01^a^
**PPPR**	0–15	21.00^a^	1.29^a^	192.00^a^	8.68^a^	3.20^a^	2.12^a^
**SED (5%)**		NS	NS	37.90	1.04	0.63	0.51
**CV (%)**		25.70	11.30	39.90	20.80	39.80	53.30
**P**	15–30	13.85^b^	1.51^a^	-	-	-	-
**R**	15–30	13.84^b^	1.67^a^	-	-	-	-
**PR**	15–30	13.74^b^	1.63^a^	-	-	-	-
**PPR**	15–30	22.66^a^	1.37^a^	-	-	-	-
**PPPR**	15–30	23.85^a^	1.33^a^	-	-	-	-
**SED (5%)**		1.31	NS	-	-	-	-
**CV (%)**		9.20	10.70	-	-	-	-

MC = Moisture content; BD = Bulk density; I = Cumulative infiltration amount; i = Infiltration rate; S = Sorptivity, K_o_ = Steady state infiltrability; P = sole plantain crop; R = sole rubber crop; PR = one row of plantain in two rows of rubber; PPR = two rows of plantain in two rows of rubber; PPPR = three rows of plantain in two rows of rubber; CV = Coefficient of variation; SED = Standard error of difference; Means with the same alphabet within a column are not statistically different

### Treatment effects on major soil nutrients and organic carbon

Total N was significantly higher (P ≤ 0.05) under the agroforestry PPR (0.22%) and PPPR (0.22%) cropping systems compared to the other cropping systems (PR, P and R) at the Ellembelle study site. The lowest total N value (0.18%) was recorded under the sole rubber (R) cropping system. At the Jomoro study site, there was no significant difference (P > 0.05) in the total N under the various cropping systems. The total mean nitrogen content across the various cropping systems at the Ellembelle site was 0.20% while that of the Jomoro site was 0.14% (Table 2A). This trend was similar to the initial soil total N at 0–15 cm where Ellembelle study site had 0.30% compared to the Jomoro site with 0.15%. The mean nitrogen was higher at the Ellembelle site than the Jomoro site.

Soil organic carbon SOC was significantly higher (P ≤ 0.05) under the PPR (2.47%) and PPPR (2.71%) cropping systems (Table 2) than the PR, P and the R systems at the Ellembelle. The lowest value (1.92%) was obtained under the sole rubber system (R) and the highest value (2.71%) was recorded under the PPPR cropping system. At the Jomoro site, there was no significant difference in the SOC under the various cropping systems. The SOC increased significantly with increasing plantain planting density in the intercrops at the Ellembelle study sites. The mean SOC under the treatments at the Ellembelle site was higher (2.33%) than that of the Jomoro site (1.76) (Table 2). Generally, the agroforestry systems (PR, PPR, and PPPR) improved the SOC content compared to the sole cropping systems (R, P).

The effect of the treatments on the soil available P content at 0–15 cm is shown in Table 3. At the Ellembelle site, the addition of plantain to rubber plantation significantly (P ≤ 0.05) increased soil available phosphorus content with PPPR obtaining a significantly highest soil available P values. The soil available P was generally low but lowest under the sole plantain cropping system (1.51 mg/kg) and relatively higher under the PPPR system (3.72 mg/kg). Mean soil available P content was 2.46 mg/kg. At the Jomoro site, soil available P was negligible and could not be detected by Bray’s no 1 extraction procedure at all sampling depths (0–60 cm). This could be attributed to excessive P fixation and complexation. The soil pH under the cropping systems was strongly acidic [17] classification. There were no significant differences in the soil pH under the various cropping systems at both the Jomoro and Ellembelle study sites (Table 3). The mean pH under the cropping systems was higher at the Jomoro site than the Ellembelle site.

**Table 3:** Effect of the treatments on major soil nutrients and organic carbon in three (0–15 cm; 15–30 cm; 30–60 cm) depths at the study sites.

Treatment	Ellembelle site					Jomoro site				
	Avail. P (mg/kg)	Total N (%)	Exch. K (cmol/kg)	SOC (%)	pH	Avail. P (mg/kg)	Total N (%)	Exch. K (cmol/kg)	SOC (%)	pH
**0–15 cm**										
**P**	1.51^b^	0.20^b^	0.18^a^	2.24^d^	4.91^a^	NP	0.14^a^	0.27^a^	1.75^a^	5.00^a^
**R**	1.69^b^	0.18^c^	0.17^a^	1.92^e^	4.90^a^	NP	0.16^a^	0.28^a^	1.90^a^	4.94^a^
**PR**	1.78^b^	0.19^bc^	0.16^a^	2.30^c^	4.73^a^	NP	0.15^a^	0.24^a^	1.80^a^	5.05^a^
**PPR**	3.61^a^	0.22^a^	0.17^a^	2.47^b^	4.92^a^	NP	0.14^a^	0.27^a^	1.71^a^	5.13^a^
**PPPR**	3.72^a^	0.22^a^	0.20^a^	2.71^a^	4.54^a^	NP	0.13^a^	0.31^a^	1.62^a^	5.04^a^
**SED (5%)**	0.60	0.01	NS	0.06	NS	NP	NS	NS	NS	NS
**CV (%)**	29.90	6.00	10.90	3.40	5.40	NP	17.80	28.00	16.00	1.30
**15–30 cm**										
**P**	3.79^a^	0.14^a^	0.14^a^	1.53^b^	4.92^a^	NP	0.09^a^	0.15^a^	1.31^a^	4.82^a^
**R**	0.12^b^	0.11^a^	0.15^a^	1.28^d^	4.56^ab^	NP	0.09^a^	0.23^a^	1.02^b^	4.70^a^
**PR**	0.13^b^	0.12^a^	0.09^b^	1.42^c^	4.74^a^	NP	0.08^a^	0.14^a^	1.00^b^	4.82^a^
**PPR**	0.16^b^	0.13^a^	0.14^a^	1.51^b^	4.62^a^	NP	0.09^a^	0.16^a^	1.06^b^	4.81^a^
**PPPR**	3.84^a^	0.14^a^	0.13^a^	1.78^a^	4.53^ab^	NP	0.08^a^	0.16^a^	1.00^b^	4.76^a^
**SED (5%)**	0.37	NS	0.02	0.06	0.34	NP	NS	NS	0.09	NS
**CV (%)**	28.00	13.80	16.10	4.60	8.90	NP	14.20	37.50	10.80	2.90
**30–60 cm**										
**P**	11.00^a^	0.04^a^	0.08^a^	0.50^b^	4.82^a^	NP	0.08^a^	0.14^a^	1.08^a^	4.62^a^
**R**	12.65^a^	0.05^a^	0.07^a^	0.54^b^	4.48^a^	NP	0.06^a^	0.14^a^	0.70^a^	4.50^a^
**PR**	11.41^a^	0.05^a^	0.08^a^	0.52^b^	4.84^a^	NP	0.07^a^	0.12^a^	0.81^a^	4.72^a^
**PPR**	10.16^a^	0.06^a^	0.08^a^	0.67^a^	4.92^a^	NP	0.07^a^	0.09^a^	0.77^a^	4.42^a^
**PPPR**	9.94^a^	0.05^a^	0.08^a^	0.64^a^	4.83^a^	NP	0.07^a^	0.13^a^	0.86^a^	4.60^a^
**SED (5%)**	NS	NS	NS	0.04	NS	NP	NS	NS	NS	NS
**CV (%)**	21.70	14.10	8.80	8.40	3.58	NP	9.80	25.30	21.10	4.40

NP = No available P in the soil, P = sole plantain crop; R = sole rubber crop; PR = one row of plantain in two rows of rubber; PPR = two rows of plantain in two rows of rubber; PPPR = three rows of plantain in two rows of rubber; CV = Coefficient of variation; SED = Standard error of difference; Means with the same alphabet within a column are not statistically different

The results presented in Table 3 showed that total N at 15–30 cm depth was not significantly influenced by cropping systems at both the Ellembelle and Jomoro study sites. The total N under the cropping systems was higher (0.13%) at the Ellembelle site than the Jomoro site (0.09%).

Soil organic carbon (SOC) differed significantly (P ≤ 0.05) among the various cropping systems at both Ellembelle and Jomoro study sites. At the Ellembelle site, the highest SOC (1.78%) was obtained from PPPR cropping system whiles the lowest was recorded under the sole rubber system. At the Jomoro site, the highest SOC was obtained under the sole plantain cropping system (1.31%) with the lowest (1.0%) from the PR and PPPR treatments. The mean SOC was higher at the Ellembelle site (2.33%) compared to the Jomoro site which has a value of 1.07%.

Available soil P was significantly (P ≤ 0.05) influenced by the different cropping systems at the Ellembelle study site. The highest value of 3.84 mg/kg was obtained from the PPPR and the lowest (0.12 mg/kg) from R cropping system. Similar to the observation made in the 0–15 cm depth, results showed that available soil P was not detected at 15–30 cm under the different cropping systems at the Jomoro site. The soil pH was significantly influenced by cropping systems at the Ellembelle but not at the Jomoro site. The highest pH value under the cropping systems at the Ellembelle study site was obtained from the sole plantain treatment (P). The mean pH was similar under the cropping systems at Jomoro than Ellembelle (Table 3).

The total soil N and available P at 30–60 cm depth were not affected by different cropping systems at the Ellembelle and Jomoro study sites (Table 3). Soil Organic Carbon (SOC) at 30–60 cm depth ranged from 0.50 to 0.67% at Ellembelle and from 0.70 to 1.08% at Jomoro under the various cropping systems (Table 3). Significant differences were observed among the SOC contents under the various systems at the Ellembelle site but the difference observed at Jomoro site was not significant. The highest SOC content was found under the PPR system and the lowest under the sole plantain system. The mean SOC was higher at the Jomoro site compared to the Ellembelle site (Table 3). Soil pH was not significantly different under the cropping systems at both study sites, with pH values ranging from 4.42 to 4.92. Soil pH was uniformly distributed under the different cropping systems at both study sites in the 30–60 cm.

### Soil microbial biomass C, N and P

Soil microbial biomass (SMB) C, N and P were significantly affected in both sites under the various cropping systems (Table 4). Presented in Table 5 is a summary of the microbial quotient and microbial carbon/nitrogen ratio under the various treatments. The observed ratios were highest under the PPPR and P at the Ellembelle and Jomoro sites. The variations among these ratios under the various systems are as a result of the differences in C_mic_. The highest soil microbial biomass C (C_mic_) was recorded under the PPPR system in both Ellembelle and Jomoro sites. The lowest C_mic_ were recorded under the sole rubber vegetation in Ellembelle and sole plantain systems in Jomoro. The C_mic_ increased with increased plantain planting density at both Ellembelle and Jomoro study sites. As with C_mic_, soil microbial biomass nitrogen (N_mic_) and phosphorus (P_mic_) were highest under PPPR in Ellembelle and Jomoro. From the results, P_mic_ was very low in Jomoro (< 10 mg/kg) compared to Ellembelle.

**Table 4:** Soil microbial properties under the various treatments at the study sites.

Treatment	Microbial biomass (mg/kg)					
	Ellembelle site			Jomoro site		
	C_mic_)	N_mic_	P_mic_	C_mic_	N_mic_	P_mic_
**P**	107.20^b^	28.76^d^	59.50^c^	38.90^c^	33.00^d^	1.95^c^
**R**	41.70^d^	11.23^e^	47.50^d^	39.50^c^	69.80^b^	3.06^a^
**PR**	94.400^c^	24.64^c^	55.50^c^	42.70^c^	55.30^c^	1.20^d^
**PPR**	104.30^b^	30.66^b^	71.80^b^	60.00^b^	71.80^b^	2.72^b^
**PPPR**	139.90^a^	36.26^a^	87.60^a^	78.70^a^	80.30^a^	3.45^a^
**SED (5%)**	4.98	1.77	6.06	4.19	4.31	0.43
**CV (%)**	6.30	8.30	11.50	9.90	8.50	21.20

C_mic_ = Microbial biomass carbon; N_mic_ = Microbial biomass nitrogen; P_mic_ = Microbial biomass phosphorus; SOC = Soil organic carbon; P = sole plantain crop; R = sole rubber crop; PR = one row of plantain in two rows of rubber; PPR = two rows of plantain in two rows of rubber; PPPR = three rows of plantain in two rows of rubber; CV = Coefficient of variation; SED = Standard error of difference; Means with the same alphabet within a column are not statistically different

**Table 5:** Microbial quotient and microbial C/N ratio.

	Ellembelle site		Jomoro site	
Treatment	C_mic_/SOC	C_mic_/N_mic_	C_mic_/SOC	C_mic_/N_mic_
**P**	0.48^a^	3.73^a^	0.22^c^	1.18^a^
**R**	0.22^c^	3.71^a^	0.21^c^	0.57^c^
**PR**	0.41^b^	3.83^a^	0.24^c^	0.77^b^
**PPR**	0.42^b^	3.40^a^	0.35^b^	0.84^b^
**PPPR**	0.52^a^	3.86^a^	0.49^a^	0.98^a^
**SED (5%)**	0.04	NS	0.06	0.24
**CV (%)**	4.64	1.30	3.45	2.24

C_mic_ = Microbial biomass carbon; N_mic_ = Microbial biomass nitrogen; P_mic_ = Microbial biomass phosphorus; SOC = Soil organic carbon; P = sole plantain crop; R = sole rubber crop; PR = one row of plantain in two rows of rubber; PPR = two rows of plantain in two rows of rubber; PPPR = three rows of plantain in two rows of rubber; CV = Coefficient of variation; SED = Standard error of difference; Means with the same alphabet within a column are not statistically different

### Plant carbon and nutrient composition

Though soil total N and SOC was higher at Ellembelle site compared with Jomoro site, assessment of the crop parameters revealed similar (P > 0.05) productivity at both sites. Plant tissue analysis was conducted on various parts of plantain and rubber to ascertain the concentrations of some macronutrients as a results of the treatments (Tables 6 and 7)

**Table 6:** Carbon and nutrient contents of plantain under the different cropping systems at Jomoro.

Agronomic parameter	Plantain			
	C (%)	N (%)	P (%)	K (%)
Young Roots	45.82±0.00	0.51±0.08	0.09±0.02	2.81±0.13
Young Stems	47.95±1.21	1.02±0.01	0.18±0.03	3.97±0.30
Young Leaves	50.27±0.67	2.48±0.14	0.29±0.02	2.28±0.41
Mature roots	43.70±3.19	0.76±0.08	0.12±0.01	1.94±0.11
Mature stems	49.11±2.34	0.64±0.09	0.09±0.01	3.41±0.11
Mature leaves	50.77±0.89	2.20±0.35	0.14±0.02	2.05±0.11

C = Carbon, N = Total nitrogen; P = Available phosphorus; K = Exchangeable potassium

**Table 7:** Carbon and nutrient contents of rubber tree under the different cropping systems at Jomoro.

Treatment	Leaves				Stem				Roots			
	C	N	P	K	C	N	P	K	C	N	P	K
**R**	54.33^a^	4.19^a^	0.47^a^	1.64^a^	51.62^d^	0.74^a^	0.08^c^	1.08^a^	51.82^c^	1.07^a^	0.12^b^	0.98^a^
**PR**	54.52^a^	4.27^a^	0.46^a^	1.68^a^	53.17^c^	0.79^a^	0.08^c^	1.11^a^	52.78^b^	0.93^a^	0.12^b^	0.83^a^
**PPR**	54.14^a^	4.02^a^	0.49^a^	1.68^a^	54.72^b^	0.71^a^	0.10^b^	1.04^a^	52.78^b^	1.01^a^	0.12^b^	0.92^a^
**PPPR**	54.91^a^	4.14^a^	0.48^a^	1.53^a^	55.49^a^	0.83^a^	0.16^a^	1.20^a^	53.75^a^	1.00^a^	0.13^a^	0.91^a^
**SED (5%)**	NS	NS	NS	NS	0.69	NS	0.01	NS	0.43	NS	0.006	NS
**CV (%)**	1.00	5.90	7.30	4.20	1.60	10.60	12.30	11.70	1.00	9.40	2.30	9.10

C = Carbon, N = Total nitrogen; P = Available phosphorus; K = Exchangeable potassium; P = sole plantain crop; R = sole rubber crop; PR = one row of plantain in two rows of rubber; PPR = two rows of plantain in two rows of rubber; PPPR = three rows of plantain in two rows of rubber; CV = Coefficient of variation; SED = Standard error of difference; Means with the same alphabet within a column are not statistically different

## Discussion

### Soil hydro-physical properties

The results revealed that cropping systems had no significant effect on bulk density for the different sampling depths, however, high bulk density values were observed under the PPPR and R systems in the 0–15 and 15–30 cm depths at Jomoro, and PPPR in both 0–15 and 15–30 cm depths at Ellembelle (Table 2). The relatively high bulk densities observed under these systems could be attributed to the corresponding low organic matter contents [18] as evidenced by the low SOC values (Table 2). According to Jiregna et al. [19], bulk density is highly influenced by tree based systems and management practices that enhance the accumulation of organic matter to modify soil properties such as bulk density. This could have possibly resulted from the swelling and sealing of the soil surface as a result of intense rains coupled with the effect of tillage operations. On the other hand, the high bulk density values recorded in PPPR (0–15 cm) and R (15–30 cm) systems at the Jomoro site are evidence of the low SOC contents coupled with very high clay contents, especially in the 15–30 cm depth.

The results showed that cropping systems had no significant effect on bulk density (Table 2). This could be attributed to the immature rubber trees (2 years) at the time of this study. Several studies have reported the effect of matured trees on reduction in soil bulk density [20, 21]. Fisher [22] also reported that N-fixing, deep rooting, and heavy litter tree species reduced soil bulk density when compared with pasture treatments. The relatively high soil bulk density observed under some of the treatments (Table 2) indicates the occurrence of soil compaction, which directly implies decreased soil porosity and reduced permeability [23]. According to Hajabbasi et al. [24], high bulk density could reduce the soil quality. Since the rubber trees in this study are young (2 years old), their impact on deeper soil horizons are yet to be expressed. Therefore, it is expected that, as the trees mature and their roots occupy greater soil volume, there will be drastic alterations in the soil pore structure in both the shallow and deeper horizons.

The different rubber and plantain cropping system did not significantly influence soil moisture content (SMC) at both sites in the 0–15 cm depth, however, significant differences were observed in the 15–30 cm depth (Table 2). This could be partly due to loss of soil water through evaporation because of open canopy [18, 25]. At both study sites, high SMC values were observed under the PPPR system. This could be attributed to the effects of litter fall [18], and the dense canopy cover highly contributed by the foliage of plantain, which provided good ground surface cover, resulting in reduced evapotranspiration rate, and runoff as evidenced by the high hydraulic properties (i.e., cumulative infiltration amount, sorptivity, infiltration rates and steady state infiltrability). This is a clear evidence that agroforestry systems could be more effective in the conservation of soil moisture through supply of litter to cover soil surface and the effects of the canopy cover.

Generally, the plantain based cropping systems promoted the movement of water in the soil as evidenced by the high cumulative infiltration amount, infiltration rate, sorptivity and steady state infiltrability values at both study sites (Table 2). This implies that plantain roots had very significant effects on the soil pore structure. The high cumulative infiltration amount at Jomoro was obtained, basically as a result of the high sorptivity (3.20 cm/min^1/2^), which controls the initial stage of infiltration (in this case, 5 min), and the steady state infiltrability (2.12 cm/min), which describes the infiltration rate toward saturation of the soil. The high water penetration rate in the soil observed under the PPPR, PPR and PR systems are clear indications that the rubber in agroforestry systems have considerable effects on both bio-physical and chemical processes that affect soil health [26]. Thus, the roots of the rubber trees coupled with those of the plantain, as well as the high sand contents within the 0–15 cm depth might have improved the soil macroporosity [27, 28], which greatly influences soil permeability. The predominance of macropores in relation to the micropores, in spite of the high bulk density values are a reflection of the observed high hydraulic properties in the study. These observations support earlier assertions on the effects of intercropping on soil, which include reduction of runoff and/or erosion, basically through the improvement of soil physical properties such as structure, porosity, and moisture retention as a result of extensive root system and the canopy cover [29].

### Major soil nutrients and organic carbon

From the study, the intercropping systems (PR, PPR, and PPPR) generally resulted in increased N and SOC contents than the sole cropping systems (R and P). These findings are in agreement with Barua and Haque [30], who reported that the SOC content and storage under intercropping systems is significantly higher than those in the open land. The general increase in N contents in the intercropping systems compared to the monocultural systems are evidence of enhanced N cycling as reported by Kumar [31] and Mbow et al. [32]. Similarly, Richard [33] reported higher N mineralization potential in tree-based intercropping systems compared to other conventional agricultural systems. This implies that the rubber trees in the various intercropping systems used in this study served as effective traps for atmospheric dust, and also acted as central points for attracting soil micro and macro fauna, for enhanced organic matter decomposition [34]. Thus, in this study, intercropping rubber with plantain, therefore, enhanced soil nutrient pools such as P, total N and SOC [35]. The observation regarding K indicates that it was taken up due to higher plant root density [36] and may have accumulated in the plant biomass (Table 5) [37].

Comparing the individual agroforestry systems, the soil pH in the different depths did not differ from the initial values in both study sites. The differences in pH observed at different soil depth at both sites could be as a result of leaching and lack of mixing of the soil profile, and variations in the soil fractions and SOC contents at different depths [38]. The relatively low pH observed under the sole rubber vegetation could be attributed to the low SOC compared to the rubber-plantain systems [39]. On the other hand, the increased accumulation of aboveground biomass and associated cation uptake in the agroforestry systems could also explain the low pH in the soils, which could probably be due to the tree root abundance in the soils resulting in high uptake of cations [36, 37].

### Soil microbial biomass C, N and P

The agroforestry systems had significant effect on soil microbial biomass C, N and P among in both the Ellembelle and Jomoro sites (Table 4). Consistent with the report by Djagbletey [40], C_mic_ and N_mic_ were significantly variable among the various treatments at both sites [38, 41, 42]. The P_mic_ differed significantly across the intercropping systems at both Ellembelle and Jomoro study sites. However, compared with other studies, such as Vance et al. [43] for temperate forest soils; Luizao et al. [44] for Amazon Basin soils, and Tornquist et al. [37] for humid tropical soils in Costa Rica, the microbial biomass in this study were considerably low. The observed differences in C_mic_ under the different cropping systems in both sites could be attributed to variable microclimates resulting from the differences in vegetation cover and actively growing vegetation [40, 45], especially in the systems involving plantain. Accordingly, Djagbletey [40] reported that larger pool of C_mic_ under vegetation could create and enhance soil physical structure with a concomitant effect on carbon sequestration. However, the buildup of C_mic_ in the PPPR systems could subsequently result in increased fluxes of trace gases from microbial processes [40, 46, 47].

The results showed that increasing vegetation cover through increased plantain population influenced both N_mic_ and P_mic_ in soils. The PPPR systems in both sites had the highest N_mic_, which implies a corresponding higher nitrification rate than the other cropping systems [40]. According to Templer [48], this could severely affect N retention in soil due to leaching. However, the high plantain density in the PPPR may counterbalance the effect of leaching since root abundance would be increased under this system. Similarly, the highest P_mic_ was observed under PPPR systems in both study sites. This suggests the potential contribution of effective agroforestry systems in soil microbial buildup. The observed high soil microbial biomass (SMB) in the 0–15 cm depth supports the findings of higher microbial biomass in the upper soil layer [40, 49]. Yan et al. [50], reported high C_mic_ under wheat crop rows in an intercropping system in China. Generally, differences in litter quantity and quality in these systems could have resulted in the significant differences in the soil microbial biomass. The high SMB associated with plantain fields could be attributed to the high litter production by plantain than rubber.

### Microbial quotient and microbial C/N ratio

The amount of metabolic active C in the total soil organic matter (SOM) as a result of the various cropping systems was described using the microbial quotient (C_mic_/SOC ratio) [40; 51–53]. Thus, the C_mic_/SOC ratio was used in this study as an indicator of the efficiency in the utilization of organic substrates by microbes [40, 54], and also to determine the sustainability of the intercropping systems, since it is generally considered as a sensitive indicator of SOM quality [55]. From this study, changes in SMB due to different agroforestry systems were faster than changes in SOC after 18 years of straw incorporation as observed by Powlson et al. [56]. Thus, the C_mic_/SOC ratio can be significantly enhanced by SOM management, improving soil microbial characteristics and slowly increasing SOC [57]. Hence, Powlson et al. [56] and Wardle [58] reported that SMB and C_mic_/SOC ratio can be effectively used as early signs of soil quality degradation. Amongst the various cropping systems, the lowest value was found under sole rubber (R) at both sites, indicating that the size of C_mic_ as a proportion of the total SOC is greater under the plantain vegetated systems (P, PR, PPR and PPPR), with PPPR having the highest. The C_mic_/SOC ratios under the various cropping systems at both study sites were generally low when compared to the critical threshold of 2.0 [40, 54], which is an indication of disturbed turnover of SOM [53, 59].

The soil C_mic_/N_mic_ ratios estimated were used as indicator of N supply ability, and also to describe the structure and state of the microbial community under the different intercropping systems [40]. Compared to other studies, the ratios reported in this study are low. For instance, Joergensen [60] reported C_mic_/N_mic_ ratios of 5.2 in an arable land, and 20.8 in a forest soil. Djagbletey [40] also reported mean C_mic_/N_mic_ ratios ranging from 5.9 to 17.7 under three different forest covers in a Savanna ecosystem in Ghana. In their study, Logah et al. [61] also found C_mic_/N_mic_ ratios ranging from 3.9 to 35 in an agricultural land in the semi-deciduous forest zone of Ghana. The low ratios observed in this study are a clear evidence of the low N contents [17] observed under the various cropping systems, and are far below that reported at a global scale, which ranges between 6 and 8 [62, 63]. According to Berg and McClaugherty [64], the C/N ratio of soil litter ranges from 12–80, which implies that microorganisms responsible for the decomposition of litter are usually faced with the challenge of excess C relative to N [65]. The low C_mic_/N_mic_ ratios observed in this study could also be attributed to the substrate imbalance owing the presence of woody plants (rubber trees), which have extremely high C/N ratio, up to about 400 [65–67].

## Conclusions

High levels of microbial biomass C and N were observed in the high plantain density plots. Similar to C_mic_ and N_mic_, levels P_mic_ also increased with decreasing plantain density. Evidently, with C_mic_, N_mic_ and P_mic_ being significantly higher under the plantain-based systems suggests that distribution pattern of soil microbial biomass under the different cropping systems was likely governed by plantain vegetation density. Microbial quotient and microbial biomass C/N ratio were efficient in the determination of the differences among various intercropping systems in the study. The results would contribute to the knowledge on flow of carbon and energy through soil microbial biomass under different agricultural land use systems.

With regard to the soil hydro-physical properties, greater improvements were observed under the rubber-plantain intercropping systems than the monocropping systems. Additionally, the study has revealed that plantain has the ability to improve soil physical and hydraulic parameters through surface cover by litter fall and canopy cover, and also root coverage in the soil. This study has revealed the potential of agroforestry under rubber to present an opportunity in increasing land productivity and improve soil fertility. In addition, agroforestry practices can increase the nutrient cycling, control of surface runoff and/or erosion, and C sequestration.

## Supporting information

S1 Data Research data. (RAR)
